# Moving towards the detection of frailty with biomarkers: A population health study

**DOI:** 10.1111/acel.14030

**Published:** 2023-12-08

**Authors:** Lana Sargent, Mike Nalls, Andrew Singleton, Priya Palta, Anna Kucharska‐Newton, Jim Pankow, Hunter Young, Weihong Tang, Pamela Lutsey, Amy Olex, Jered M. Wendte, Danni Li, Alvaro Alonso, Michael Griswold, B. Gwen Windham, Stefania Baninelli, Luigi Ferrucci

**Affiliations:** ^1^ Virginia Commonwealth University School of Nursing Richmond Virginia USA; ^2^ Department of Pharmacotherapy and Outcomes Science, Geriatric Pharmacotherapy Program, School of Pharmacy Virginia Commonwealth University Richmond Virginia USA; ^3^ National Institutes of Health, Center for Alzheimer's and Related Dementias National Institute of Aging Bethesda Maryland USA; ^4^ Data Tecnica International Glen Echo Maryland USA; ^5^ Department of Neurology University of North Carolina at Chapel Hill School of Medicine Chapel Hill NC USA; ^6^ Department of Epidemiology, College of Public Health University of Kentucky Lexington Kentucky USA; ^7^ Memory Impairment and Neurodegenerative Dementia Center University of Mississippi Medical Center Jackson Mississippi USA; ^8^ Welch Center for Epidemiology, Prevention, and Clinical Research Johns Hopkins University Bloomberg School of Public Health Baltimore Maryland USA; ^9^ Division of Epidemiology and Community Health, School of Public Health University of Minnesota Minneapolis Minnesota USA; ^10^ Division of Epidemiology and Community Health School of Public Health Minneapolis Minnesota USA; ^11^ C. Kenneth and Dianne Wright Center for Clinical and Translational Research Virginia Commonwealth Univerity Richmond Virginia USA; ^12^ Department of Lab Medicine and Pathology University of Minnesota Minneapolis Minnesota USA; ^13^ Department of Epidemiology, Rollins School of Public Health Emory University Atlanta Georgia USA; ^14^ Laboratory of Clinical Epidemiology, InCHIANTI Study Group Local Health Unit Tuscany Center Florence Italy; ^15^ Longitudinal Studies Section, Translational Gerontology Branch National Institute on Aging Baltimore Maryland USA

**Keywords:** biomarkers, frailty, machine learning

## Abstract

Aging adults experience increased health vulnerability and compromised abilities to cope with stressors, which are the clinical manifestations of frailty. Frailty is complex, and efforts to identify biomarkers to detect frailty and pre‐frailty in the clinical setting are rarely reproduced across cohorts. We developed a predictive model incorporating biological and clinical frailty measures to identify robust biomarkers across data sets. Data were from two large cohorts of older adults: “*Invecchiare in Chianti* (Aging in Chianti, InCHIANTI Study”) (*n* = 1453) from two small towns in Tuscany, Italy, and replicated in the Atherosclerosis Risk in Communities Study (ARIC) (*n* = 6508) from four U.S. communities. A complex systems approach to biomarker selection with a tree‐boosting machine learning (ML) technique for supervised learning analysis was used to examine biomarker population differences across both datasets. Our approach compared predictors with robust, pre‐frail, and frail participants and examined the ability to detect frailty status by race. Unique biomarker features identified in the InCHIANTI study allowed us to predict frailty with a model accuracy of 0.72 (95% confidence interval (CI) 0.66–0.80). Replication models in ARIC maintained a model accuracy of 0.64 (95% CI 0.66–0.72). Frail and pre‐frail Black participant models maintained a lower model accuracy. The predictive panel of biomarkers identified in this study may improve the ability to detect frailty as a complex aging syndrome in the clinical setting. We propose several concrete next steps to keep research moving toward detecting frailty with biomarker‐based detection methods.

## Graphical abstract

The frailty phenotype is a clinically recognizable validated measure of changes in body composition, compromised energetics, and homoeostatic decompensation. We used a complex systems approach to biomarker selection to examine frailty biomarker population differences. The study results represent further advancements in biomarker‐based research for detecting frailty as a complex aging syndrome. The graphical abstract will be submitted with the final version of the article.

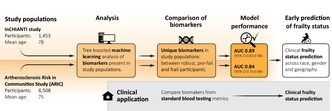

**Abbreviations**
ACB: Anticholinergic Cognitive Burden
ALT: alanine aminotransferase
ARIC: Atherosclerosis Risk in Communities Study
AUC: area under the curve
CES‐D: Center for Epidemiologic Studies Depression Scale
CHS: Cardiovascular Health Study
FN: false negatives
FP: false positives
HR: hazard ratios
IL‐6: interleukin‐6
InCHIANTI Study: Invecchiare in Chianti
ML: machine learning
ROC: receiver operating characteristic
TN: true negatives
TNFR 1 & 2: soluble TNF‐a receptor I and II
TP: true positives

## 1 INTRODUCTION

There have been significant changes to the age demographics in the United States, with Americans 65 years and older projected to reach more than 22% of the total population by 2050 (Day, 2011). The U.S. aging population is becoming more diverse by race, with Asians being the fastest‐growing population (US Census Bureau, 2020), and the Black population growing to 59 million by 2050, a 56% rise over four decades (Day, 2011; US Census Bureau, 2020; Vincent & Velkoff, 2010). An expanding aging population has brought a concurrent rise in the number of older adults with frailty (Rohrmann, 2020; Yu et al., 2018). Frailty is one of the most significant challenges for healthcare professionals caring for aging populations due to the increased likelihood of unmet care needs, including hospitalizations, falls, and early mortality (Dent et al., 2019; Hoogendijk et al., 2019; Mazya et al., 2019; Mocchegiani et al., 2012). Assessment of frailty is useful to prognosticate risk and determine individuals who may benefit from interventions and those for which burdensome treatments should be avoided.

Frailty measures health vulnerability and compromised ability to cope with routine or acute stressors (Fried et al., 2001; Makary et al., 2010). The frailty phenotype is a clinically recognizable validated measure of changes in body composition, compromised energetics, and homoeostatic decompensation (Fried et al., 2001; Makary et al., 2010). It is associated with increased dependency and adverse health outcomes, including high hospital readmission and postoperative mortality rates (Macdonald et al., 2021; Makary et al., 2010; Mocchegiani et al., 2012). Perioperative pre‐frail and frail older adult patients have a 2.54 times higher odds of longer length of stay or greater likelihood of being discharged to a skilled or assisted‐living facility when compared to non‐frail older adults (Makary et al., 2010; Mohanty et al., 2016). This increased risk spurred a joint statement from the American College of Surgeons and the American Geriatrics Society in 2012 recommending a frailty assessment as a part of the preoperative evaluation for all older adults (Makary et al., 2010). Subsequently, the Society for Perioperative Assessment and Quality Improvement outlined practical steps for clinicians to assess frailty in older adults who require elective intermediate or high‐risk surgery (Mohanty et al., 2016). Urgency in the need for early recognition and interventions for frailty has been recognized as a public health priority by the World Health Organization (Anon, 2015).

Due to the heterogeneity in the presentation of frailty, especially in the pre‐frail stages, it is often difficult for clinicians to recognize, manage, and treat frail patients. Clinicians strive to do what works best within a system where providers are overwhelmed with caring for multiple complex diseases, often in patients with complicated health disparities (Cardoso et al., 2018). Encouraging clinical guidelines for frailty screening is imperative now that growing numbers of studies have demonstrated interventions that can improve frailty biomarkers and reverse frailty scores (Hsieh et al., 2019; Jha et al., 2017; Mazya et al., 2019; Sadjapong et al., 2020; Tarazona‐Santabalbina et al., 2016). For those whom interventions are ineffective, it is essential to avoid harm by recommending interventions that would not improve health and could shorten life or worsen the quality of life.

Despite evidence that frailty screening effectively identifies patients at the highest risk for adverse outcomes in medical and surgical specialties, assessing frailty in clinical settings has been problematic for several reasons. After over 20 years of research, there is no universally accepted reference standard, nor do we have established predictive biological markers to guide clinicians in the early detection or prevention of frailty (Bergman et al., 2007; Panza et al., 2011). Multiple operational definitions have been suggested, and numerous functional tests, questionnaires, and indexes are available (Bergman et al., 2007). This has led to confusion among clinicians and a lack of utility for screening in clinical practice. Other limitations include the time or special equipment required to complete the frailty screening instruments, which can hinder providers under pressure to maintain productivity (Munyon et al., 2017). When selecting a frailty instrument, clinicians consider various factors, such as the instrument's validity across settings, time available in the clinical setting, and the purpose of screening. Over 16 primary frailty instruments are available with five scales: the Frailty phenotype (Fried et al., 2001; Makary et al., 2010), Frailty index (Rockwood et al., 1999), Clinical Frailty (Church et al., 2020), FRAIL scale (Morley et al., 2012), and Study of Osteoporotic Fractures frailty criteria (Ensrud et al., 2008) validated across the primary care, hospital, and long‐term care facility settings (Church et al., 2020). Yet, none of the clinical screening instruments allow for the detection of early biological changes to detect the pre‐frail and frail stages. It is essential to capture biological risk factors as early as possible to intervene before symptoms of frailty arise, leading to further decline and loss of independence (Dent et al., 2019).

The combination of frailty measurement tools and biomarker detection would complement the frailty detection (Cardoso et al., 2018). In a multisystem syndrome such as frailty, it is essential to note that biomarkers have multiple physiological roles and may relate to the causal mechanism, resilience response, or simply proxy biomarkers. The mechanistic nature of biomarkers included in screening and diagnostic tools does not affect their performance for case findings. A model using biomarker associations as a proxy for detecting vulnerability could provide practitioners with the tools needed for the early detection of individuals with frailty. Studies have identified individual frailty biomarkers, such as inflammatory responses, hormones, and free radicals going back to 2002 (Ferrucci et al., 2002), including combining endocrine and inflammatory markers as frailty predictors (Puts et al., 2005). However, many of the proposed clinical biomarkers of frailty are often not reproduced across various cohorts. This study addresses this gap by developing a predictive model incorporating the top predictive biological and clinical measures and verifies the findings across population health studies. We propose several concrete next steps to keep research moving toward detecting frailty with biomarker‐based detection methods.

## 2 METHODS

### 2.1 Study characteristics

**FIGURE 1:**
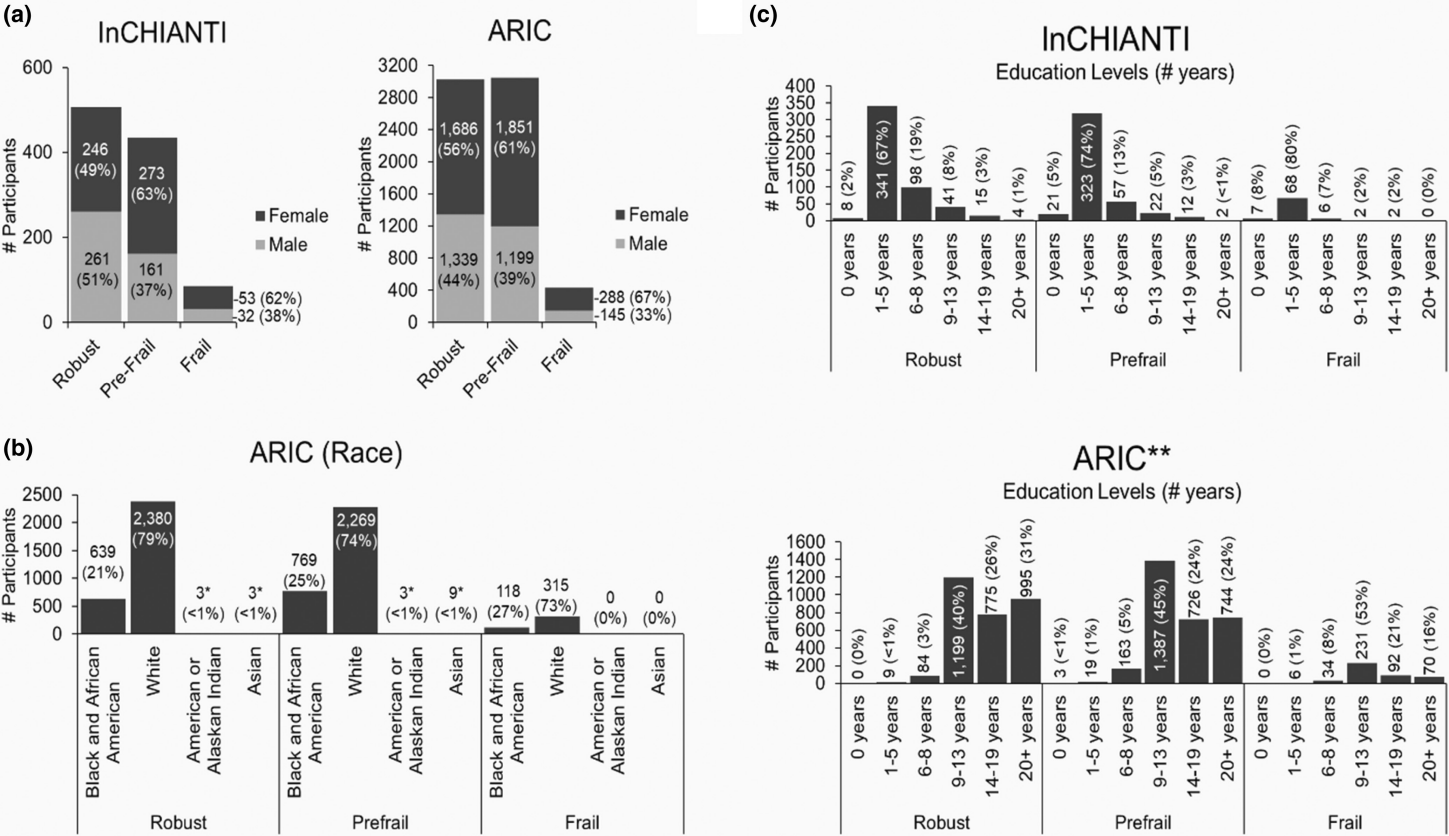
Study population demographics. (a) Female vs. male participants. (b) Race distribution in the ARIC study. The InCHIANTI study is a White European population demographic. *American Indian or Alaskan Indian and Asian populations were not included in the study due to small sample sizes. (c) Education levels. **The ARIC study was missing education information for 3 individuals classified as Robust and 8 individuals classified as Pre‐frail.

Data were from the “*Invecchiare in Chianti* (Aging in Chianti, InCHIANTI Study”) with a representative sample (*n* = 1453, mean age = 78, 64%women) of older adults from two small towns in Tuscany, Italy, and replicated in the Atherosclerosis Risk in Communities Study (ARIC) (*n* = 6508, mean age = 75, 58% women, 23% Black) at the fifth exam comprising older adults from four U.S. communities (Forsyth County, North Carolina; Jackson, Mississippi; Minneapolis, Minnesota; and Washington County, Maryland) (Figure 1). Frailty category distributions for InCHIANTI were 507 (49%), 434 (42%), and 85 (8%) for robust, pre‐frail, and frail, respectively, and 3025 (46%), 3050 (46%), and 433 (7%) for each category, respectively, for ARIC. Overall, a major portion of the InCHIANTI study participants had 1–5 years of education, compared to the majority of ARIC participants having 9+ years of education (Figure 1). The lower education levels in the InCHIANTI are characteristic of the older adult population in this rural region at the time of the study. The current study used biomarker and frailty data from the InCHIANTI baseline (1997–1989) through Visit 5 (2011–2015) and frailty assessment at ARIC Visit 5 (2011–2013), which contained the earliest frailty assessment in ARIC.

### 2.2 Study design

We utilized clinical biomarkers in epidemiological aging research to identify a cluster of biomarker proxies, rather than a single biomarker, that represent the complex system changes to identify frailty status reliably. This study used a complex systems approach to biomarker selection and an epidemiological methodologic approach to ensure proper biomarker inquiry for aging research (Cohen et al., 2018). Our previous work informed the biomarkers, which were tested in association with clinical outcomes using a tree‐boosting, machine learning (ML) technique for supervised learning analysis (Sargent et al., 2018; Sargent, Nalls, Amella, Slattum, et al., 2020). We examined biomarker population differences across and within the datasets. Predictors found with robust, pre‐frail, and frail participants from the InCHIANTI data were replicated using ARIC data. We explored the final model's ability to accurately detect frailty status across Black and White participants in ARIC. We consider race differences between InCHIANTI and ARIC as strengths rather than limitations, allowing for external replication in different cohorts.

### 2.3 Measures

*Frailty phenotype*: Frailty, as defined by the Cardiovascular Health Study (CHS), allows for an ordinal scoring system versus a nominal system because it can capture the multidimensional nature of frailty: robust, pre‐frail, and frail participants (Fried et al., 2001; Hirsch et al., 2006). InCHIANTI and ARIC studies used frailty as defined by the CHS with the following domains: weight loss, low physical activity, low grip strength, slow walking speed, and exhaustion (Ferrucci et al., 2000; Kucharska‐Newton et al., 2017). The frailty phenotype is defined in three categories—robust (0 criteria), pre‐frail (1–2 criteria), and frail (3–5 criteria) (Fried et al., 2001; Rockwood et al., 1999). InCHIANTI and ARIC frailty components have concurrent and predictive validity with hazard ratios (HR) ranging from 1.82 to 4.46 (*p* < 0.05) for outcomes that include incident disease, hospitalization, falls, disability, and mortality in community‐dwelling older adults (Fried et al., 2001; Kucharska‐Newton et al., 2017; Stenholm et al., 2018). Details on the operationalization of frailty for comparison across the InCHIANTI and ARIC studies compared to the original frailty definition in the CHS can be found in Table S1 (Ferrucci et al., 2000; Kucharska‐Newton et al., 2017; Stenholm et al., 2018).

*Anticholinergic burden calculation*: Our team's previous research conducted in InCHIANTI found Anticholinergic Cognitive Burden (ACB) is a strong predictor of physical frailty (Sargent, Nalls, Amella, Mueller, et al., 2020). This study used the ACB scale, a validated scale for assessing adverse health outcomes associated with anticholinergic burden, including cognitive and physical function (Church et al., 2020; Morley et al., 2012). The anticholinergic properties of each medication were quantified using the ACB scale based on each drug's serum anticholinergic activity (Collamati et al., 2016). To determine ACB scores, each medication was assigned points (0, 1, 2, 3) according to the published 2012 update and summed for a total anticholinergic burden score (Mueller et al., 2020; Stewart et al., 2021). Higher scores indicate higher anticholinergic properties. Examples of medications with ACB scores include Nortriptyline = 3, Cyclobenzaprine = 2, and Nifedipine = 1. *Depression score*: The CES‐D self‐report scale (0–60) measures depressive symptoms. Reliability, validity, and factor structure have been similar across diverse demographics, and the scale has been used extensively in epidemiologic studies for depressive symptoms and physical function (Arts et al., 2015; Lewinsohn et al., 1997; Perna et al., 2017). *Demographics*: Age at the time of assessment is used as a continuous variable, and race and ethnicity were self‐reported. American Indian or Alaskan Indian (*n* = 6) and Asian (*n* = 12) were removed from the analysis due to the small numbers represented in the frailty groups (Figure 1). *Biomarkers*: A complete list of all the biomarker variables used in the model, including laboratory measures of inflammation and clinical measures of anticholinergic burden, can be found in Table S2.

### 2.4 Analysis and workflow

**FIGURE 2:**
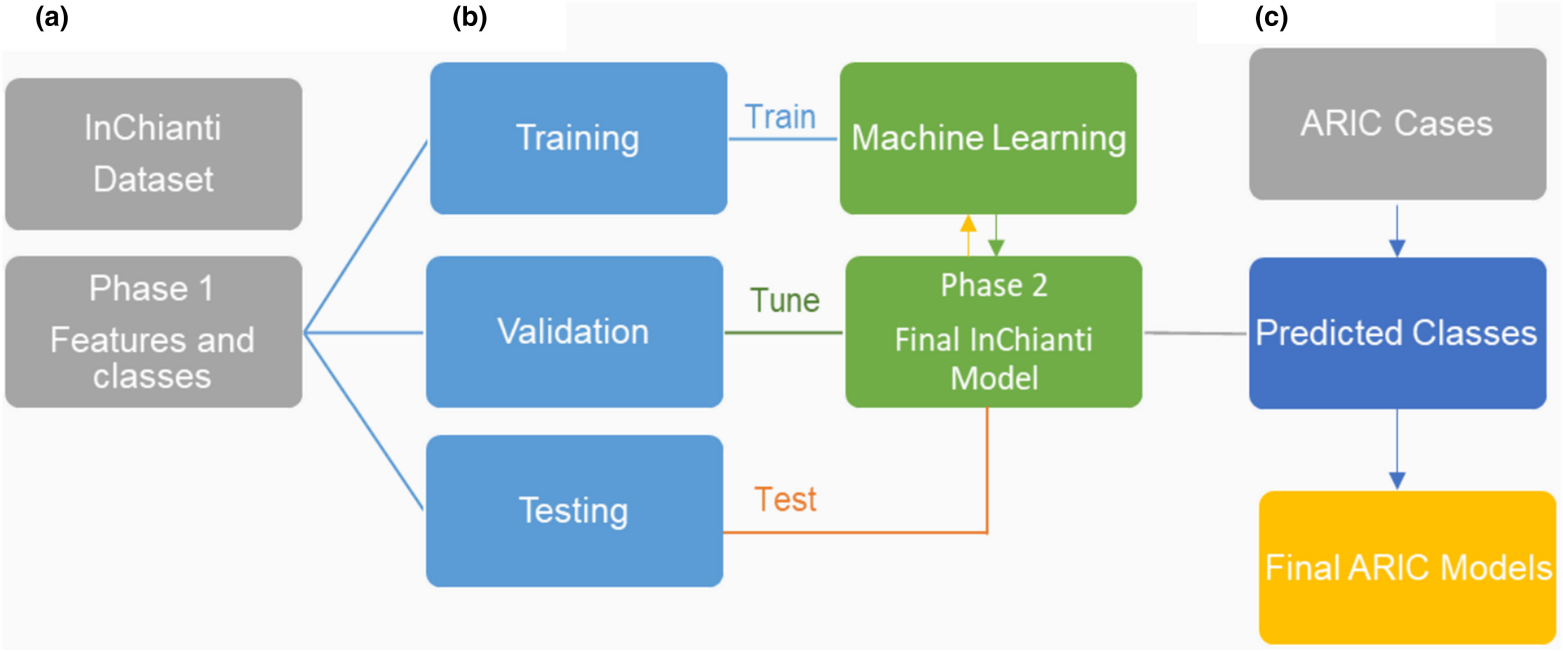
Study workflow overview of the predictive machine learning model. (a) The predictive clinical and laboratory biomarkers were extracted in phase 1 (b) training was used to select the model hyperparameters and a test set to evaluate the performance of the final model. k‐fold cross‐validation was applied to each problem's data, extending the holdout method until in phase 2 we achieved model performance for prediction of frailty groups. (c) Findings were replicated in the ARIC cohort to test model accuracy.

The statistical analysis was completed in the RStudio software package using R 4.1.2. using the following steps: Phase one of the workflow included (1) data preprocessing, reduction, and analysis of all available variables for biomarker feature selection, (2) model training, validation, and performance, and (3) determination of the significance in the models' features. Phase two included using the top predictive features from Phase One of the workflow to test the model's prediction accuracy in ARIC. Figure 2 highlights the general workflow and study approach diagram as described above. Additional details for the model generation and calibration of the model can be found in the Appendix S1.

Our study used the boosted tree approach for data pruning, classification, and regression tree algorithms with hyperparameters set for each problem. The advantage of using a tree‐boosting approach model for evaluating multiple variables simultaneously is that it provides a high predictive value with a low bias (Chen & Guestrin, 2016). A gradient‐boosted trees method builds a more accurate classifier model by repeatedly reweighting the training examples, improving upon the regression model; then, the final model uses withheld test samples to evaluate the prediction model. The hyperparameters were retained to create the best‐performing model and then used to retrain the model and on the complete data to develop the final model. This process determined the accuracy of classifying patients into robust, pre‐frail, and frail groups. Boosted trees used individual decision trees that account for multicollinearity between the biomarker variables, thus controlling for complex system interactions in which many of the biomarker variables are interdependent; with a statistical distance approach, the model retained only the best features in the final model (Chen & Guestrin, 2016; Markatou & Sofikitou, 2019). Covariates were selected to control for potential confounding effects, including sex, age, education, and depression.

### 2.5 Performance metrics and model evaluation

With any predictive model in ML, there is a chance for an inflated risk of capitalizing on chance features (overfitting) in the data. Overfitting of the model was mitigated in two ways: (1) having a distinct training and validation process for the model and (2) using parameter settings for selection to reduce poor predictive performance. The holdout method was used to split the datasets into training and testing randomly; in this study, we used training datasets (70%) and evaluated test datasets (30%). The training datasets were used to build the model, while the test dataset was used to assess prediction capabilities. A k‐fold cross‐validation procedure was applied to each problem's data, extending the holdout method by repeating the splitting process several times. We used 5‐fold cross‐validation, training to select model hyperparameters, and a test set to evaluate the performance of the final model, maintaining the ratio of the classes while doing the 5‐fold validation. The scale_pos_weight hyperparameter was implemented to scale the gradient for the positive classes (pre‐frail and frail) relative to the control (robust). This was an essential preprocessing step to handle imbalanced data and helped the model achieve better performance when making predictions of the positive class (pre‐frail and frail). Using standardized beta‐coefficients allowed comparisons of the relative effect sizes of predictors measured on different scales. The Hosmer‐Lemeshow test assessed the goodness of fit (Lemeshow & Hosmer, 1982; Nalls et al., 2015). We used the evaluation metrics receiver operating characteristic (ROC) curve and area under the curve (AUC) to evaluate the models' performance. AUC was calculated from each model to determine the discrimination of participants with frail (case) from robust (control) in the training cohort. An AUC of 0.5 was considered chance, >0.8 informative, and >0.9 clinically relevant (Li & He, 2018). Next, we evaluated the results of the models to correctly predict frailty groups from robust groups using additional performance measures formulated using the true positives (TPs), False positives (FPs), true negatives (TNs), and false negatives (FNs).

### 2.6 Feature selection

The predictive clinical and laboratory biomarkers were identified in Phases 1 and 2 using the InCHIANTI data. One sample *t*‐test for continuous variables with a Bonferroni correction was used to determine the significance of the variables between robust and pre‐frail groups and robust and frail groups. In each binary classification model, all variables were ranked by level of importance in the model, where the best subset of the features was chosen using chi‐square feature selection. The multinomial analysis determined the ability of the final selected features to capture the progressive multidimensional nature of frailty, robust, pre‐frail, and frail groups. In the ARIC dataset, some biomarker measurements were available only at different time points than the frailty outcome measure (ARIC Visit 5). The model used data close to the outcome diagnosis (ARIC Visit‐5 Frailty) to examine the AUC. We also examined model parameters and AUCs by adding features in a stepwise process from Visits 1 through 5. As variables were added, parameters (model fit and AUC) were examined for best fit with the Delong method for confidence intervals (DeLong et al., 1988). Despite some biomarkers in ARIC being collected at different times from the frailty assessment, the findings in InCHINATI were replicated in the ARIC cohort, illustrating the replication and value of the biomarker set for future studies. Table S3 in the Appendix S1 highlights the stepwise logistic regression process used to measure varying temporal differences in the ARIC Visits 1 through 5 biomarker data. Variables were removed from the analysis if there was >15% missing data (Table S2). Because the exhaustion criterion from the physical frailty definition is derived from the depression scale CES‐D, a sensitivity analysis was performed by excluding the exhaustion criterion from the frailty definition (Raji et al., 2002). The outcomes remained statistically significant (*p* < 0.001) (Figure S1).

## 3 RESULTS

### 3.1 InCHIANTI and ARIC model results

**FIGURE 3:**
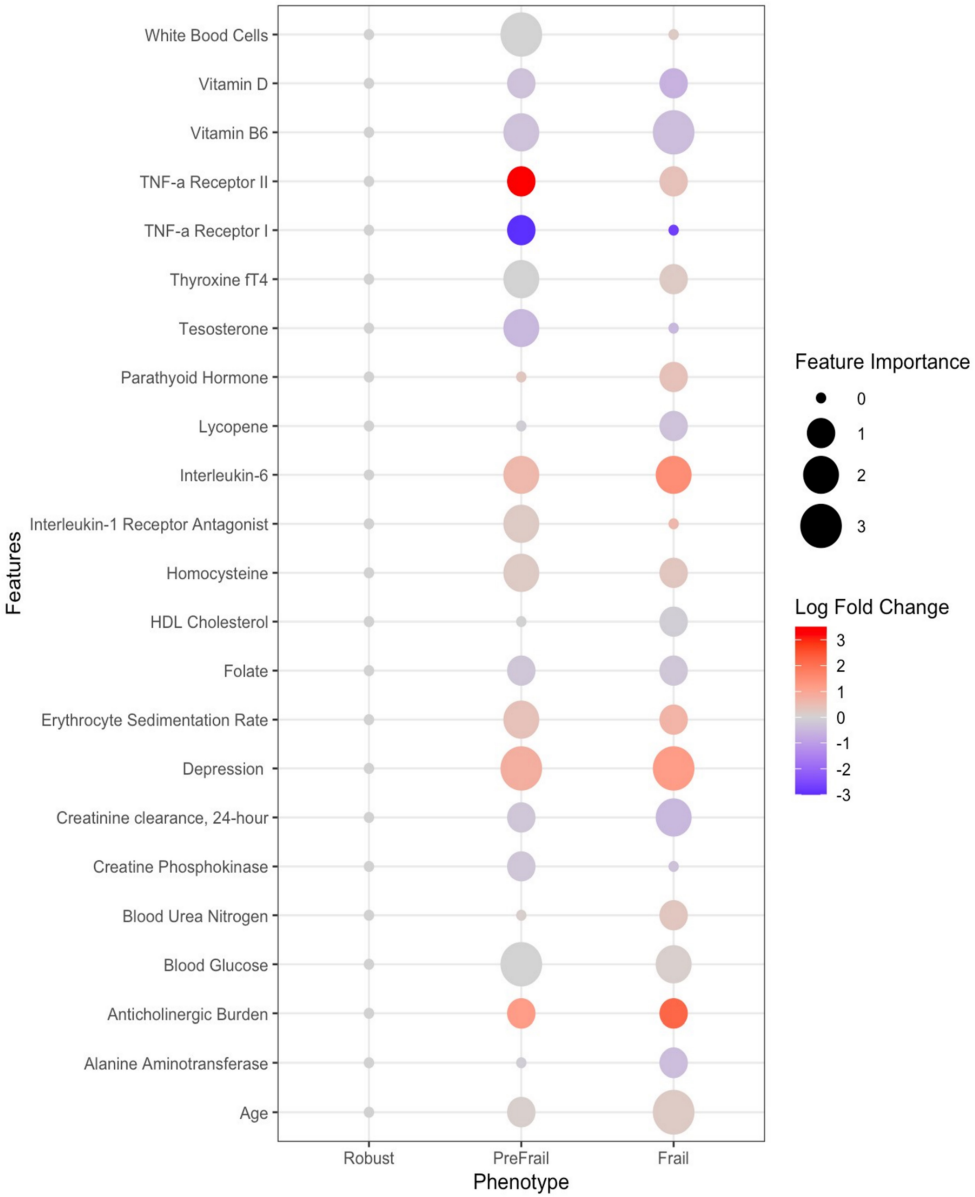
Bubble plot of the importance and log fold change by phenotypes. The size of the bubble is proportional to the importance level of the feature, the larger the bubble the greater effect the feature has on predicting the phenotype. The log fold change becomes negative when the mean value of the feature decreases and positive when the mean value increases.

Age and sex distributions across population health studies are similar, with mean age ranges of 72–81 years and females representing a more significant proportion of the population in all categories. Race and ethnicity varied across studies due to the sample populations of the studies; InCHANTI contains a White European population, and ARIC represents a population of mostly Black and White participants in the United States. Education years varied across studies, with a mean of 5.3 years (3.3 SD) for InCHANTI and 15.1 years (4.3 SD) in the ARIC population (Figure 1). A total of 85 (8.2%) were classified as frail, 434 (42.3%) pre‐frail in InCHIANTI, 433 (6.5%) frail, and 3038 (46.8%) pre‐frail in ARIC. The binary model biomarker feature selection found 23 features with significant mean differences among robust, pre‐frail, and frail phenotypes (Table 1). Binary prediction model performance for the InCHIANTI and ARIC replication models can be found in Table S4. Multivariate classification models using the unique biomarker features (*n* = 23) identified in the InCHIANTI study resulted in an AUC of 0.89 (95% confidence interval (CI) 0.82–0.98) and a model accuracy of 0.72 (95% CI 0.66–0.80). The InCHIANTI multivariate classification model AUC improved with the addition of the final selected biomarkers (AUC (95% CI) = 0.89 (0.82–0.98)) compared to age only (AUC = 0.67 (0.42–0.72)) and age and depression symptom predictors (AUC = 0.78 (0.64–0.91)). Using available predictors in ARIC from Visits 1 through 5 (Tables S2 and S5), the multivariate prediction model maintained an AUC of 0.84 (95% CI 0.75–0.89) with a model accuracy of 0.64 (95% CI 0.66–0.72). Multivariate classification model performance metrics for InCHIANTI and ARIC populations by phenotype can be found in Table 2. The bubble plot (Figure 3) shows the patterns of importance and the log fold change for each feature by phenotype. The bubble size is proportional to the importance level of the feature; the larger the bubble size, the greater the feature's effect on predicting the phenotype. The log fold change becomes negative when the mean value of the feature decreases and positive when the mean value increases. Refer to Table 1 for the mean values of each feature.

**TABLE 1:** InCHIANTI feature selection: Predictive features by frailty status.

Features	Robust mean	SE	Pre‐frail mean	SE	Frail mean	SE	*p*‐value
^a^White blood cells (WBC) (*n*, K/μL)	6.01	0.07	6.15	0.07	7.02	0.17	<0.0001
Vitamin D (nmol/L)	55.33	1.61	45.3	1.76	37.78	3.87	<0.0001
Vitamin B6 (ng/mL)	7.5	0.36	6.27	0.39	5.82	0.89	0.0348
^a^TNF‐a receptor II (pg/mL)	2620.62	35.29	29,770.8	38.24	3319.5	89.3	<0.0001
^a^TNF‐a receptor I (pg/mL)	13,338.64	29.61	1679.16	32.08	2110.2	81.3	<0.0001
Free thyroxine, fT4 (ng/dL)	1.47	0.01	1.48	0.02	1.62	0.04	<0.0001
^a^Free testosterone (ng/dL)	2.42	0.09	1.72	0.1	1.68	0.22	<0.0001
Parathyroid (pg/mL)	23.91	0.93	29.7	1.02	30.93	2.35	<0.0001
^b^Lycopene (Âμmol/L)	0.71	0.01	0.66	0.01	0.59	0.03	0.0098
^a^Interleukin‐6 (pg/mL)	1.65	0.18	2.55	0.2	4.61	0.45	<0.0001
^a^Interleukin‐1 (pg/mL)	142.21	5.2	165.95	5.65	215.56	12.8	<0.0001
^a^Homocysteine (Âμmol/L)	14.92	0.28	16.61	0.31	18.17	0.63	<0.0001
HDL cholesterol (mg/dL)	56.67	0.67	55.3	0.73	52.82	1.66	0.0545
Folate (ng/mL)	3.49	0.09	3.06	0.1	2.97	0.23	0.0027
Erythrocyte sedimentation rate (mm/h)	17.82	0.81	24.32	0.89	29.01	2.05	<0.0001
^a^Depression CES‐D self‐report scale	9.22	0.34	15.56	0.39	20.95	0.89	<0.0001
Creatine phosphokinase (U/L)	104.63	2.61	88.96	2.84	82.86	6.49	<0.0001
^a^Blood urea nitrogen (mg/dL)	33.74	0.44	36.68	0.56	40.82	1.3	<0.0001
^a^Blood glucose (mg/dL)	96	1.17	94.412	1.32	102.82	3.01	0.0283
^a^Anticholinergic burden (ACB scale)	0.45	0.06	1.00	0.06	2.00	0.11	<0.0001
Alanine aminotransferase (ALT) (U/L)	20.28	0.51	19.08	0.55	15.85	1.15	0.004
Age	72	0.3	76	0.32	80	0.82	<0.0001
24 h Urine creatinine (mg/24 h)	1023.66	14.92	887.85	16.94	741.25	37.6	<0.0001

*Note*: *p*‐value indicates a significant difference by frailty status.

^a^ Clinically significant change noted by accepted biological variable reference ranges.

^b^ Clinical reference range has not been established.

**TABLE 2:** Frailty Multivariate Model Performance for InCHIANTI and ARIC.

Measure	InCHIANTI			ARIC		
	Robust	Pre‐frail	Frail	Robust	Pre‐frail	Frail
AUC 95% CI	0.89 (0.82–0.98)			0.84 (0.75–0.89)		
Model accuracy 95% CI	0.72 (0.66–0.80)			0.64 (0.66–0.72)		
Sensitivity %	75.6	53.0	97.8	65.7	49.7	82.8
Specificity %	83.8	84.4	90.7	79 0.8	79.9	89.0
Positive predictive value % (precision)	72.1	65.0	84.6	62.9	55.3	78.3
Negative predictive value %	82.4	79.6	98.7	81.7	76.1	91.5

**FIGURE 4:**
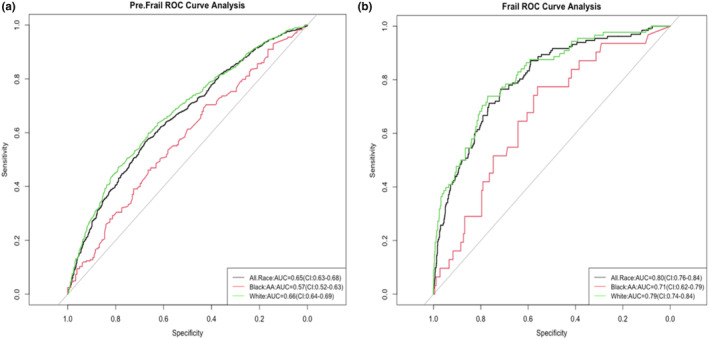
ROC Curve Pre‐frail and Frail Phenotypes Across Race Models in ARIC. The final model's ability to detect frailty status across races in ARIC for (a) pre‐frail and (b) frail phenotypes.

Delong's test for two ROC curves highlights the model fit by phenotype and race (Figure 4). The final model's ability to accurately detect frailty status across races in ARIC resulted in differences for pre‐frail and frail groups by race. There are fewer frail Black individuals *n* = 118 than White *n* = 315, similar to pre‐frail groups by race *n* = 769 and *n* = 2269, respectively. The frail model found significant differences between the two models: (1) the all‐race population model and Black population model (*p* = 0.02), and (2) the White population model and Black population model *p* = 0.04. No difference was found between the all‐race and White population model *p* = 0.56. The pre‐frail model Delong's test for two ROC curves (Figure 4) found similar results; all‐race population model and Black population model *p* < 0.01, White population model and Black population model *p* < 0.01, and no difference between all‐race and White population models *p* = 0.43.

*Clinical markers*: Depressive symptoms were one of the top predictors for both pre‐frail and frail status, with an increase in depression symptoms in frail individuals. As expected, age increased from robust (72 years), pre‐frail (76 years), to frail (80 years), with a progression from less important to most important in predicting phenotype status. Anticholinergic drug burden maintained a level of importance across both phenotypes (*p* < 0.0001), with higher levels of drug burden in the frail phenotype. *Markers of inflammation*: higher inflammation was seen with increasing mean levels across phenotypes of erythrocyte sedimentation (*p* < 0.0001), interleukin‐6 (*p* < 0.0001), homocysteine (*p* < 0.0001), and with lower levels of soluble TNF‐a receptor I and II (sTNFR1 & 2) activity (*p* < 0.0001). *Markers of metabolic and endocrine function: Decreased* levels of metabolic function were observed with lower mean levels across phenotypes with creatine phosphokinase and 24‐h urine creatinine (*p* < 0.0001), with 24‐h creatinine clearance being a strong predictor in frail patients. Serum creatinine was not retained as an essential feature in the final models. Free thyroxine, fT4 was higher (*p* < 0.0001), and alanine aminotransferase (ALT) was lower in frail individuals (*p* < 0.0001) compared to robust and pre‐frail levels. *Nutrient and lipid metabolism*: Vitamin B6, Folate, and D deficiency were progressively lower across groups of frail status (*p* < 0.0001). Vitamin D deficiency decreases from pre‐frail to frail status, with increased parathyroid levels becoming a more accurate predictor in the frail group (*p* < 0.0001).

## 4 DISCUSSION

A predictive model using population health data to determine the top predictive features will help identify frail individuals or those at risk for frailty. The resulting models use the top predictive biomarkers and clinical data to show reliable predictive power. In addition, our results show significant performance by reducing the variables in the model. Replicating the prediction model in ARIC maintained predictive function (84%); however, overall model accuracy decreased (64%). There is a low model performance for predicting pre‐frail status across studies; this may be related to the heterogeneity in the pre‐frail stages and the loss of significant features in the final model due to missing data. The prediction of frail status from robust status had the highest sensitivity and specificity, likely representing progression toward homogeneity in the phenotype.

Anticholinergic drug burden is a novel clinical marker for predicting frailty phenotypes and may reflect the progressive disease burden and polypharmacy seen in the later stages of life. However, increased levels of anticholinergic drug burden further compound morbidity and mortality (Collamati et al., 2016; Jamsen et al., 2016). High levels of anticholinergic drug burden in frail individuals can lead to poor health outcomes such as delirium and worsening cognitive outcomes (Ah et al., 2019; Mueller et al., 2020).

This study's proposed panel of biomarkers is verified across frailty cohorts and correlates with biological markers described in over 10 years of previous biomarker frailty research. In particular, inflammatory and metabolic markers such as IL‐6 and TNFR 1 & 2 are associated with physical performance, gait speed, and progressive depressive symptoms (Arts et al., 2015; Brown et al., 2016). ALT is an enzyme that helps break down proteins into energy and is a marker of decreased energy expenditure for frail adults with sarcopenia (Vespasiani‐Gentilucci et al., 2018). Lower than normal ALT levels often indicate vitamin B6 deficiency and chronic kidney disease, also found as significant predictors in pre‐frail and frail models.

### 4.1 Strengths and limitations

Deep phenotyping for frailty allowed the analysis of race (Black and White) in InCHIANTI and ARIC. However, larger numbers of frail individuals will be needed to refine the prediction of frailty across race and ethnic populations, including Black, Asian, and Hispanic or Latino frail individuals, as our cohorts lacked large enough numbers to include race and ethnic representation other than Black and White participants. Furthermore, we could not distinguish regional influences as Black participants in ARIC were primarily from a single US site. Frailty variations may serve as a marker for differences in the frequency of genetic polymorphisms that affect biomarkers such as inflammation (Barbato et al., 2004; Hirsch et al., 2006). Future systems models could include mixed‐effect longitudinal disease progression models and unsupervised ML modeling across harmonized data. These methodologies may test the assumptions in this study's biomarker and clinical features. The boosted trees method in this study harnessed individual decision trees to account for multicollinearity between the variables, thus allowing us to control for biomarker variables interdependence; with a statistical distance approach, the model retained only the best features in the final models. The biomarker interrelationships seen in our results may represent a biological decline in the physiologic cycle of frailty.

### 4.2 Steps forward

This study considered critical epidemiological methodologic approaches to advance understanding of the physiological underpinnings of frailty using biomarkers in aging research, such as (1) replication from a White/European population in Italy in a cohort of mostly Black and White participants in the US and (2) a non‐linear methodology analysis in which individual decision trees account for multicollinearity among the biomarker variables. The study findings need further replication in a harmonized data set with increased population diversity before being translated into the clinical setting. Additional research is required in order to develop biological and clinical prediction models; data harmonization and democratization will reduce fragmented access to biological markers and allow for comprehensive analysis of aging syndromes with deep phenotyping. We should continue to support similar approaches to identifying frail individuals from administrative claims‐based and electronic medical record data. The increasing availability of large‐scale proteomics and metabolomics data across diverse ethnic/racial groups with data democratization and harmonization will be a powerful tool for improving biomarker‐based prediction models. Most importantly, the harmonization of multiple longitudinal population studies will permit analysis of multisystem dynamics in frailty progression and model the change in biomarkers through the disease progression.

## 5 CONCLUSION

The study results represent further advancements in biomarker‐based research for detecting frailty as a complex aging syndrome. Striving to produce models that facilitate appropriate identification and diagnosis to reduce the burdens for patients and providers along the diagnostic pathway is essential to progress.

## AUTHOR CONTRIBUTIONS

LS and JMW drafted the manuscript. LS, MG, LF, MN, and AO contributed to the study design and data analysis. LS, MG, LF, and GW provided data curation and project administration. The ARIC Publication Committee provided a manuscript and statistical review with formal manuscript approval. All authors contributed to the design of the study, data interpretation, and manuscript revision.

## CONFLICT OF INTEREST STATEMENT

L.F. serves on the editorial board of Aging Cell. There are no other conflicts of interest to declare.

## Supporting information


Appendix S1

## Data Availability

The analytic methods and study materials are available for reproducing the results or replicating the procedure at https://github.com/neurogenetics/Detection‐of‐Frailty‐with‐Biomarkers‐A‐Population‐Health‐Study. ARIC and InCHIANTI investigators are committed to enhancing scientific research reproducibility due to consent restrictions and recording of some variables to reduce the risk of identification of participants; the data may not be identical. Individual‐level patient data may be further restricted by consent, confidentiality, or privacy laws/considerations.
