# Supplementary material for: Moving towards the detection of frailty with biomarkers: A population health study
Source: Aging Cell. 2023 Dec 8;23(2):e14030. doi: 10.1111/acel.14030 (PMC10861189; doi:10.1111/acel.14030)
Supplement: Supplementary file 1 — Appendix S1 [file ACEL-23-e14030-s001.docx]

**Design and analysis (study design, inclusion/exclusion, outcome, and other variables of interest with specific reference to the time of their collection, summary of data analysis, and anticipated methodologic limitations or challenges).**

*The analytic methods and study materials have been available to the researcher for purposes of reproducing the results or replicating the procedure at* [*https://github.com/neurogenetics/Detection-of-Frailty-with-Biomarkers-A-Population-Health-Study*](https://github.com/neurogenetics/Detection-of-Frailty-with-Biomarkers-A-Population-Health-Study)*. ARIC and InCHIANTI investigators are committed to efforts to enhance the reproducibility of scientific research due to consent restrictions and recording of some variables to reduce the risk of identification of participants the data may not be identical. The data can be made available upon request through the ARIC and InCHIANTI investigators.*

***1. Methods/Study Design***

A cross-sectional predictive model comparing predictors with robust, pre-frail, and frail participants developed from the baseline InCHIANTI data was used to replicate a population predictive model in ARIC. The ARIC model is run separately with a comparison of the AUC. The results discuss the attributable AUC differences in part based on outcome derivations. An Extreme Gradient Boosting (xgboost) in R(Chen, Tianqi, He, Tong, Benesty, Michael, Khotilovich, Vadim, Tang, 2017) for the validation model in ARIC. While boosting was initially developed for machine learning, ‘xgboost’ in R is based on boosted trees. Xgboost is an open-source tool and a variant of the gradient boosting machine and uses a tree-based model. Xgboost is used in this study for a supervised learning problem where the variables identified are used to predict pre-frail and frail individuals. Statistical distances for model adequacy were performed in the ARIC models based on biomarker distance from the phenotype measurement V5-timepoint (S. Table 2).

1.1. Model Generation

The predictive clinical and laboratory biomarkers identified in InCHIANTI Model 1 are analyzed using an Extreme Gradient Boosting (xgboost) in R (Chen, Tianqi, He, Tong, Benesty, Michael, Khotilovich, Vadim, Tang, 2017) for the replication model in ARIC. While boosting was initially developed for machine learning, ‘xgboost’ in R is based on boosted trees. Xgboost is an open-source tool and a variant of the gradient boosting machine and uses a tree-based model. Xgboost is used in this study for a supervised learning problem where the variables identified from a previous study are used to predict pre-frail and frail individuals (Sargent et al., 2018).

1.2. Calibration of the model

Parameter estimates for each predictive factor and associated descriptive statistics will be evaluated to provide biological insight into the underpinnings of the classification algorithm. We evaluated the calibration by partitioning the data into 5, 10, 20, 30, 40, 50, 75, 100, and 200 groups and then ran the calibration test. Next, we repeated tests for all possible values between 5-200 groups and evaluated the distribution of the test statistic. The best prediction thresholds were determined using AUC. S.Table 3. Represents the univariate frailty prediction model fit InCHIANTI and ARIC for used for testing and calibration. Multivariate models used for the final prediction models (details described in the manuscript) have been saved for public use in transfer learning and are available on GitHub for this report.

1.3. Frailty Phenotype

The frailty phenotype is defined in three categories—robust (0), pre-frail (1-2), and frail (3-5) (Fried et al., 2001; Kucharska-Newton et al., 2017). The outcome measure of frailty is used from V5 in the ARIC dataset. For this study, the V5-derived variable frailty52 is used. The frailty52 variable uses a missing at random (MAR) assumption to recover information amongst those participants with some but not all of the 5 frailty characteristics. If the participant has all 5 indicators observed, they are considered frail, 3 characteristics observed; they are classified as frail, and 1-2 characteristics they are considered pre-frail. If the participant has only 4 observed characteristics, then 3-4 is considered frail, and 1-2 is considered pre-fail. Frailty52 yields a frailty sample size of *N*=433. The same protocol for deriving the frailty variables was used in InCHANTI; there were 298 that were <65 and not included. InCHANTI yields a frailty sample size of *N*=85. ARIC contains a frailty51 variable at V5 derived, which does not use the missing at random (MAR) assumption. The composite frailty is classified by the frailty characteristics and the same number of components, but participants with missing information on all component characteristics were classified as missing the frailty phenotype yielding a frailty sample size of *N*=368 (Kucharska-Newton et al., 2017).

1.4. Predictors

1.4.1. Anticholinergic Burden Calculation

The Anticholinergic Cognitive Burden (ACB) scale is the most clinically validated scale for evaluating adverse health outcomes, including cognitive and physical function (Campbell et al., 2010; Salahudeen et al., 2015). The anticholinergic properties of each medication will be quantified using the ACB scale based on each drug’s serum anticholinergic activity (Cai et al., 2013). ACB scores for each participant’s medications will be assigned points (0, 1, 2, 3) according to the published 2012 update and summed for a total anticholinergic burden score. Higher scores indicate higher anticholinergic properties. An example of medications with ACB scores include Amitriptyline = 3, Amantadine = 2, and Atenolol = 1.


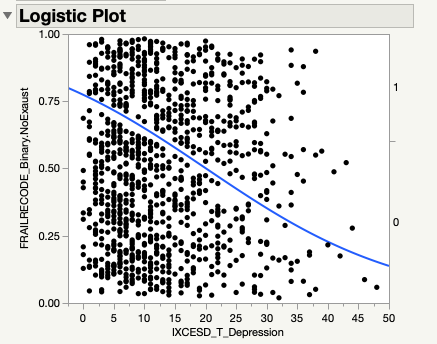


S.Figure 1. Frail No Exhaustion Regression Plot

1.4.2. Depression Score

The CES-D self-report scale (0-60) is used to measure depressive symptoms. Reliability, validity, and factor structure have been similar across a diverse demographic, and the scale has been used extensively in epidemiologic studies for depression and physical function (Radloff, 1977). The CES-D score will be used in the predictive model. Because the exhaustion criterion from the physical frailty definition is derived from the depression scale CES-D, a sensitivity analysis was performed by excluding the exhaustion criterion from the frailty definition. The frailty scores were recalculated on the other 4 frailty components, and the analyses were repeated. The final outcomes remained statistically significant, p-value < .001 (S.Figure 2.).

1.4.3. Table 1. represents the validated biological and clinical markers used to predict frailty in the InCHIANTI data and the clinical and biological markers available for the prediction of frailty in the ARIC dataset. In ARIC, 3 biological markers were used as substitutes (serum TNF used instead of TNF-a receptor I&II, total testosterone used instead of Free testosterone, and serum creatinine used instead of Creatinine clearance, 24-hr urine/24-hour urinary creatinine). InCHAINTI model has a total of 19 frail clinical and biological markers, and 13 total frail markers are available in ARIC. InCHAINTI model has a total of 22 pre-frail clinical and biological markers, and 16 total prefrail markers are available in ARIC.

**1.5 Potential Limitations**

1.5.1 Several of the biomarker measurements come from different time points than the outcome measure of frailty at V5. The model is built with data as close to the outcome diagnosis (V5 Frailty) as possible. Additionally, we examine model parameters and AUC, adding variables from visits 1 through 5. As variables were added, parameters (model fit and AUC) were examined for best fit. Initial rebuild in InCHIANTI with the predictors from the ARIC model maintained an AUC of .897. Although we cannot completely control for the varying temporal differences between some predictors and the frailty outcome, we find the findings to be informative and useful for future biomarker-based modeling studies.

1.5.2. Frailty measure differs slightly between databases. Often there are variations in how frailty is measured between longitudinal studies, which may affect the AUC and the ability of the InCHIANTI model to “fit” or accurately predict frailty in the ARIC data. However, frailty in ARIC was based on the same frailty phenotype that is used in InCHIANTI and has been validated.

1.5.3. Demographics and ethnic and racial differences between databases. The InCHIANTI database is a geographically homogeneous population with a large White European population. We consider it a strength rather than a limitation that ARIC is a more diverse population and will allow external validation to be conducted in different cohorts. Models explored ethnic and racial differences in the model fit as the model was being built.

References

Cai, X., Campbell, N., Khan, B., Callahan, C., & Boustani, M. (2013). Long-term anticholinergic use and the aging brain. *Alzheimer’s and Dementia*, *9*(4), 377–385. https://doi.org/10.1016/j.jalz.2012.02.005

Campbell, N. L., Boustani, M., Lane, K., Gao, S., Hendrie, H., Murrell, J., Unverzagt, F., Hake, A., Smith-Gamble, V., & Hall, K. (2010). Use of anticholinergics and the risk of cognitive impairment in an African-American population. *Neurology*, *75*, 152–159.

Chen, Tianqi, He, Tong, Benesty, Michael, Khotilovich, Vadim, Tang, Y. (2017). *’xgboost’-Extreme Gradient Boosting*. https://doi.org/10.1145/2939672.2939785>

Fried, L. P., Tangen, C. M., Walston, J., Newman, A. B., Hirsch, C., Gottdiener, J., Seeman, T., Tracy, R., Kop, W. J., Burke, G., & McBurnie, M. A. (2001). Frailty in older adults: evidence for a phenotype. *The Journals of Gerontology. Series A, Biological Sciences and Medical Sciences*, *56*(3), M146-56.

Kucharska-Newton, A. M., Palta, P., Burgard, S., Griswold, M. E., Lund, J. L., Capistrant, B. D., Kritchevsky, S. B., Bandeen-Roche, K., & Windham, B. G. (2017). Operationalizing Frailty in the Atherosclerosis Risk in Communities Study Cohort. *The Journals of Gerontology: Series A*, *72*(3), 382–388. https://doi.org/10.1093/GERONA/GLW144

Radloff, L. S. (1977). A Self-Report Depression Scale for Research in the General Population. *Appl. Psychol. Meas.*, *1*(3), 385–401. https://doi.org/10.1177/014662167700100306

Salahudeen, M. S., Duffull, S. B., & Nishtala, P. S. (2015). *Anticholinergic burden quantified by anticholinergic risk scales and adverse outcomes in older people: a systematic review*. https://doi.org/10.1186/s12877-015-0029-9

Sargent, L., Nalls, M., Starkweather, A., Hobgood, S., Thompson, H., Amella, E. J., & Singleton, A. (2018). Shared biological pathways for frailty and cognitive impairment: A systematic review. *Ageing Research Reviews*, *47*, 149–158. https://doi.org/10.1016/J.ARR.2018.08.001

S.Table 1. Operationalization of the Frailty Construct Across Population Health Studies

| Characteristics of Frailty | InCHIANTI(*N*=1,026) | ARIC (N=6,508) | CHS(*N*=5,317) |
| --- | --- | --- | --- |
| Weight loss | Weight loss was defined as a weight loss of greater than 5% in the previous 3 years. In the InCHIANTI study weight loss at baseline was defined based on question regarding weight loss during the past 12 months | 10% of weight loss from V4 (1996-1998) to V5 (2011-2013) or BMI <18.5 at Visit 5 | Lost > 10 lbs in last year |
| Low physical activity | Physical activity level in last year was assessed during the home interview. Low physical activity was defined as sedentary state or performing light intensity activity (i.e., walking) less than 1 hour a week | Gender-specific 20^th^ percentile rang of the Baecke leisure sports activity index | 270 on an activity scale (18 items) |
| Slow walking speed | Walking speed was measured by a 4-m walking test with a usual speed. Slow walk was defined by the lowest quintile, stratified by sex and height | Gender- and height-adjusted time in seconds used to walk 4 m, converted from CHS 15 feet walk thresholds for height and sex; Men Height ≤ 173cm, > 173cm; Women ≤ 159cm, >159cm | Walking 15 ft stratified by height and sex; height and sex; Men Height ≤ 173cm, > 173cm; Women ≤ 159cm, >159cm |
| Exhaustion | Two items from the CES-D scale: Responded “some of the time” or “most of the time” to the following questions: *I felt everything I did was an effort or I could not get “going”* | Two items from the CES-D scale Responded “some of the time” or “most of the time” to either of the following questions: *I felt everything I did was an effort or I could not get “going”* | Two items from the CES-D scale Responded “some of the time” or “most of the time” to following questions: *I felt everything I did was an effort or I could not get “going”* |
| Low grip strength | Gender- and BMI specific grip strength the maximal value of two measurements from the best hand was used and weakness was defined as by the lowest quintile | Gender- and BMI-specific grip strength in the lowest 20% percentile of CHS distributions  ARIC and CHS are same*:  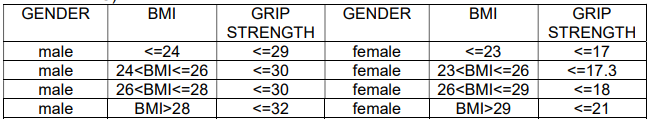 | Gender- and BMI-specific grip strength in the lowest 20% percentile of CHS distributions  *stratified by sex |

“*Invecchaiare in Chianti* (Aging in Chianti, InCHIANTI Study”), Atherosclerosis Risk in Communities Study (ARIC), Cardiovascular Heart Study (CHS)

S.Table 2. Biological and Clinical Predictors: InCHIANTI and ARIC (supplemental table)

|  | **InCHIANTI** n=1,026  Baseline Data | **ARIC** | | |
| --- | --- | --- | --- | --- |
| **Clinical** | Variable | Variable | n | Time Point |
| Age | X | X | 6,508 | V5 |
| Anticholinergic Burden | X | X | 6,508 | V5 |
| Depression/CES-D self-report scale | X | X | 6,508 | V5 |
| **Inflammatory/Immunity** |  |  |  |  |
| 24-hour urinary cortisol (µg/24 hours) | X | NA | NA | NA |
| Erythrocyte sedimentation rate (ESR) (mm/hour) | X | NA | NA | NA |
| Homocysteine via FPIA analysis (Âµmol/L) | X | X | 328 | V1 |
| Interleukin-1 via ELISA (pg/mL) | X | X | 442 | V1 |
| Interleukin-6 via ELISA ultrasensitive (pg/mL) | X | X | 572 | V1 |
| Monocyte chemoattractant protein-1 via Bio-Plex (pg/mL) | X | X | 604 | V1 |
| Soluble TNF-a receptor I via quantitative sandwich EIA (pg/mL) | X | NA*  (serum TNF) | 170 | V4 |
| Soluble TNF-a receptor II via quantitative sandwich EIA (pg/mL) | X | NA*  (serum TNF) | 170 | V4 |
| **Hematology/Liver** |  |  |  |  |
| Folate via RIA (ng/mL) | X | X | 6,166 | V3 |
| Mean corpuscular volume (MCV) (fL) | X | X | 6,281 | V5 |
| BL White blood cells (WBC) (n, K/µL) | X | X | 6,443 | V5 |
| Retinol via high performance liquid chromatography (µmol/L) | X | NA | NA | NA |
| GPT (also known as ALT) (U/L) | X | X | 5,997 | V4 |
| **Endocrine/Hormones** |  |  |  |  |
| 25(OH)-D (25-hydroxyvitamin D) via RIA (nmol/L) | X | X | 6,026 | V3 |
| Free testosterone (ng/dL), Vermeulen | X | NA*  (total testosterone ng/dL) | 5,555 | V4 |
| Blood glucose (mg/dL) | X | X | 6,108 | V5 |
| Free thyroxine, fT4 (ng/dL) | X | X | 6,409 | V5 |
| Parathyroid hormone, two-site immunoradiometric assay (pg/mL) | X | X | 6,054 | V2 |
| **Metabolomics(plasma lipids)** |  |  |  |  |
| Fatty acid C24:0 weight (mg/L) | X | NA | NA | NA |
| Lipids: HDL cholesterol (mg/dL) | X | X | 6,404 | V5 |
| **Renal/Electrolyte** |  |  |  |  |
| Creatine phosphokinase (U/L) | X | NA | NA | NA |
| Creatinine clearance, 24-hr urine (mL/minute) | X | NA*  (urine creatinine) | 6,317 | V5 |
| Urine proteins (mg/dL) | X | NA | NA | NA |
| 24-hour urinary creatinine (mg/24 hours) | X | NA*  (urine creatinine) | 6,317 | V5 |
| Blood urea nitrogen (mg/dL) | X | X | 6,461 | V1 |
| **Nutrient Biomarker** |  |  |  |  |
| Vitamin B6 via high performance liquid chromatography (ng/mL) | X | X | 6,166 | V3 |
| Vitamin E gamma tocopherol, high performance liquid chromatography (Âµmol/L) | X | X | 6,166 | V3 |
| Lycopene via high performance liquid chromatography (Âµmol/L) | X | NA | NA | NA |

*substitute biomarker was used because exact biomarker match was not available, red noted >15% missing removed from model

S.Table 3. Stepwise logistic regression to measure temporal biomarker differences ARIC

| Frail Stepwise Logistic Regression | | | | |  | Pre-Frail Stepwise Logistic Regression | | | |
| --- | --- | --- | --- | --- | --- | --- | --- | --- | --- |
| V1 | **V2** | **V3** | **V4** | **V5** |  | **V1** | **V3** | **V4** | **V5** |
| AGE | AGE | AGE | AGE | AGE |  | AGE | AGE | AGE | AGE |
| BUN | BUN | BUN | BUN | BUN |  | **AUC = .60** | Folate | Folate | Folate |
| AUC = .67 | Parathyroid | Parathyroid | Parathyroid | Parathyroid |  |  | Vit D | Vit D | Vit D |
|  | **AUC = .67** | Vit D | Vit D | Vit D |  |  | Vitamin B6 | Vitamin B6 | Vitamin B6 |
|  |  | Folate | Folate | Folate |  |  | Vitamin E IU | Vitamin E IU | Vitamin E IU |
|  |  | B6 | B6 | B6 |  |  | **AUC = .60** | Testosterone | Testosterone |
|  |  | **AUC = .68** | ALT | ALT |  |  |  | **AUC = .60** | Urine Creatinine |
|  |  |  | **AUC = .69** | HDL |  |  |  |  | Depression |
|  |  |  |  | Urine Creatinine |  |  |  |  | ACB Score |
|  |  |  |  | Fasting glucose |  |  |  |  | WBC |
|  |  |  |  | T4 |  |  |  |  | **AUC = .63** |
|  |  |  |  | ACB Score |  |  |  |  |  |
|  |  |  |  | CESD |  |  |  |  |  |
|  |  |  |  | **AUC = .72** |  |  |  |  |  |

S.Table 4. Binary Frailty Prediction Models Fit InCHIANTI and ARIC

|  | **InChianti** | | |  | **ARIC** | | |
| --- | --- | --- | --- | --- | --- | --- | --- |
| **Measure** | **Robust/Pre.Frail** | **Pre.Frail/Frail** | **Frail/Robust** |  | **Robust/Pre.Frail** | **Pre.Frail/Frail** | **Frail/Robust** |
| **AUC 95% CI** | **0.77 (0.71-0.83)** | **0.74 (0.69-0.85)** | **0.95 (0.84-0.98)** |  | **0.65 (0.63-0.68)** | **0.62 (0.56-0.67)** | **0.80 (0.76-0.84)** |
| **Model Accuracy** | **0.7266** | **0.8253** | **0.8956** |  | **0.6249** | **0.6238** | **0.8726** |
| **Sensitivity** | **0.7647** | **0.5435** | **0.8696** |  | **0.5502** | **0.5455** | **0.5036** |
| **Specificity** | **0.7045** | **0.9333** | **0.8987** |  | **0.6955** | **0.6351** | **0.9443** |
| **Positive Predictive Value (Precision)** | **0.6000** | **0.7576** | **0.5556** |  | **0.6309** | **0.1769** | **0.5798** |
| **Negative Predictive Value** | **0.8378** | **0.8421** | **0.9793** |  | **0.6204** | **0.9067** | **0.9258** |
| **F1 Score** | **0.6724** | **0.6329** | **0.678** |  | **0.5878** | **0.2672** | **0.5391** |
| **Balance Accuracy** | **0.7346** | **0.7384** | **0.8841** |  | **0.6228** | **0.5903** | **0.7239** |

S.Table 4. ARIC Binary Frailty Prediction Models Fit by Race

|  | **Black/African American** | | | |  | **White** | | |
| --- | --- | --- | --- | --- | --- | --- | --- | --- |
| **Measure** | **Robust/Pre.Frail** | **Pre.Frail/Frail** | **Frail/Robust** | |  | **Robust/Pre.Frail** | **Pre.Frail/Frail** | **Frail/Robust** |
| **AUC 95% CI** | **0.60 (0.53-0.64)** | **0.57 (0.52-0.63)** | | **0.71 (0.62-0.79)** |  | **0.64 (0.61-0.67)** | **0.66 (0.64-0.69)** | **0.79 (0.74-0.84)** |
| **Model Accuracy** | **0.5672** | **0.5687** | **0.8042** | |  | **0.6332** | **0.6122** | **0.8213** |
| **Sensitivity** | **0.5732** | **0.5283** | **0.5135** | |  | **0.6407** | **0.4894** | **0.5124** |
| **Specificity** | **0.7951** | **0.6108** | **0.8571** | |  | **0.8147** | **0.7250** | **0.8764** |
| **Positive Predictive Value (Precision)** | **0.5802** | **0.5864** | **0.7958** | |  | **0.6628** | **0.6207** | **0.5001** |
| **Negative Predictive Value** | **0.7904** | **0.5536** | **0.9062** | |  | **0.7996** | **0.6070** | **0.9172** |
| **F1 Score** | **0.5767** | **0.5558** | **0.6788** | |  | **0.6516** | **0.5473** | **0.5339** |
| **Balance Accuracy** | **0.6842** | **0.5696** | **0.6853** | |  | **0.7277** | **0.6072** | **0.6445** |

S.Table 5. Frailty Model Feature Availability Across Population Health Studies

| InChianti Frailty Model Features | ARIC Available Data | ARIC Time Point |
| --- | --- | --- |
| AGE | X | V1 |
| 25(OH)-D (25-hydroxyvitamin D) | X | V3 |
| Folate via RIA | X | V3 |
| Free testosterone | X | V4 |
| Vitamin B6 | X | V3 |
| Depression CES-D Scale | X | V5 |
| Total Anticholinergic Burden | X | V5 |
| Blood glucose | X | V5 |
| Blood urea nitrogen | X | V1 |
| Free thyroxine, fT4 | X | V5 |
| GPT (also known as ALT) | X | V4 |
| Lipids: HDL cholesterol | X | V5 |
| Parathyroid hormone | X | V2 |
| White blood cells (WBC) | X | V5 |
| Creatine phosphokinase | Not Available |  |
| Erythrocyte sedimentation rate (ESR) | Not Available |  |
| Homocysteine | X* | V1 |
| Interleukin-6 | X* | V1 |
| Soluble TNF-a receptor I | X* | V4 |
| Soluble TNF-a receptor II | X* | V4 |
| 24-hour urinary creatinine | Not Available |  |
| Lycopene | Not Available |  |
| Interleukin-1 receptor antagonist | X* | V1 |

V1= Visit 1 (1987-89); V2= V2 (1990-92); V3 (1993-95); V4 (1996-98); V5 (2011-13), *biomarker with >15% missing data
